# Pullout strength of different pedicle screws after primary and revision insertion: an in vitro study on polyurethane foam

**DOI:** 10.1186/s12891-023-07015-3

**Published:** 2023-11-07

**Authors:** Lien-Chen Wu, Yueh-Ying Hsieh, Fon-Yih Tsuang, Yi-Jie Kuo, Chia-Hsien Chen, Chang-Jung Chiang

**Affiliations:** 1 Department of Orthopaedics, Shuang Ho Hospital, Taipei Medical University, New Taipei City, 23561 Taiwan; 2 Department of Orthopaedics, School of Medicine, College of Medicine, Taipei Medical University, Taipei, 11031 Taiwan; 3 Graduate Institute of Biomedical Materials and Tissue Engineering, College of Biomedical Engineering, Taipei Medical University, Taipei, 11031 Taiwan; 4 Division of Neurosurgery, Department of Surgery, National Taiwan University Hospital, Taipei City, 10022 Taiwan; 5 Spine Tumor Center, National Taiwan University Hospital, Taipei City, 10022 Taiwan; 6 Department of Orthopedic Surgery, Wan Fang Hospital, Taipei Medical University, Taipei City, 11696 Taiwan; 7 School of Biomedical Engineering, College of Biomedical Engineering, Taipei Medical University, Taipei, 11031 Taiwan

**Keywords:** Pedicle screw, Pullout strength, Screw thread, Biomechanics

## Abstract

**Background:**

Surgeons are routinely required to remove loose or failed pedicle screws and insert a new screw in their place. However, inserting a new screw into an existing hole may compromise the holding capacity of the pedicle screw. The purpose of this study is to evaluate the pullout strength of pedicle screws with different thread designs after the primary insertion and revision surgery in a synthetic bone model.

**Methods:**

Four pedicle screws with different thread designs (single-lead-thread (SLT) screw, dual-lead-thread (DLT) screw, mixed-single-lead-thread (MSLT) screw, and proximal-unthreaded-dual-thread (PUDL) screw) were inserted into pre-drilled, untapped holes (ø 4.2 mm, length 35 mm) in Sawbone blocks of density 20 pcf. In the first sequence, a 6.0 mm screw was inserted into the predrilled foam block and the primary pullout strength of the screw was measured according to ASTM F543. In the second sequence, a 6.0 mm screw was inserted and removed, and then either a 6.5 mm screw of the same design or a different screw design was inserted into the same hole and the pullout strength recorded.

**Results:**

In the first sequence, the mean pullout strength of the MSLT screw was significantly (p < 0.05) greater than all other screw designs. In the second sequence, when the MSLT screw was the primary screw, using a larger MSLT screw (6.5 mm) as the revision screw did not lead to a higher pullout strength than if a 6.0 mm diameter PUDL screw was used for the revision. Using a larger DLT screw (6.5 mm) as the revision screw resulted in a significantly (p < 0.05) greater pullout strength than a 6.0 mm STL, DLT, MSLT, or PUDL screw.

**Conclusions:**

Our results indicate that employing classic oversizing of the same screw design is a safe choice for maintaining screw purchase in the bone after revision. In cases where oversizing with the same screw design is not practical, opting for a PUDL screw with the same original diameter can provide enough purchase in the bone to maintain stability.

## Introduction

Due to the complex loading environments in the spine or in patients with compromised bone stock, surgeons may find it necessary to remove and replace failed pedicle screws. Removing a pedicle screw can substantially reduce the quality of the bone, and therefore limit the holding capacity of the replacement screw. The revision screw is typically larger than the original pedicle screw to improve purchase with surrounding bone. Theoretically, replacing a screw with one of larger diameter and longer shaft could increase the contact area between the bone and screw, resulting in a greater pullout strength due to the increased friction force [1, 2]. However, standard practice is for the surgeon to select the largest permissible screw diameter for the primary surgery, so using a larger screw for reversion surgery may compromise the pedicle bone, leading to further loss of fixation and neural injury [3].

Alternatively, pedicle screws augmented with bone cements such as polymethylmethacrylate (PMMA) or calcium phosphate can also improve the fixation strength [4, 5]. However, there have been reports of ischemia and radicular irritation due to the heat given off from freshly injected cement [6, 7]. In addition, asymptomatic cement leakage has been reported in up to 66.7% of patients treated with augmented pedicle screws [6]. Hence, the use of cement augmented pedicle screws is still controversial.

Recent studies have investigated the relationship between the thread design on the screw and its pull-out strength [8–10]. Weegens et al. [8] reported that using a pedicle screw with a dual thread design for revision surgery can offer a similar failure strength to a traditional single thread pedicle screw that is one size larger than the original screw. However, in contrast, Seng et al. [10] found that a dual thread design could not provide a comparable pullout strength to a larger diameter screw. Such inconsistencies in the reported effects of thread design on the screw failure strength indicate there is no consensus on the best approach to revision surgery. The purpose of this study is to evaluate how using a different thread design between primary surgery and revision surgery affects the failure strength of the screw fixation in a synthetic bone block. The aim is to understand what combination of thread designs for primary and revision surgery provides the greatest pullout strength, which may improve the longevity of revision surgeries.

## Materials and methods

### Synthetic bone model

Rigid polyurethane foam blocks (Grade 20 (0.32 g/cm^3^) (1522-03; Sawbones, Pacific Research Laboratories Inc, WA, USA)) were used as a substitute for cadaveric bone because of the consistent material properties, homogenous structure, and ready availability [11]. A density of 0.32 g/cm^3^ was chosen based on the results of previous literature showing similar compressive mechanical properties with vertebral cancellous bone [12]. The foam type and representative bone densities correspond to the requirements of ASTM F1839-08 [13].

### Pedicle Screw Design

**Fig. 1:**
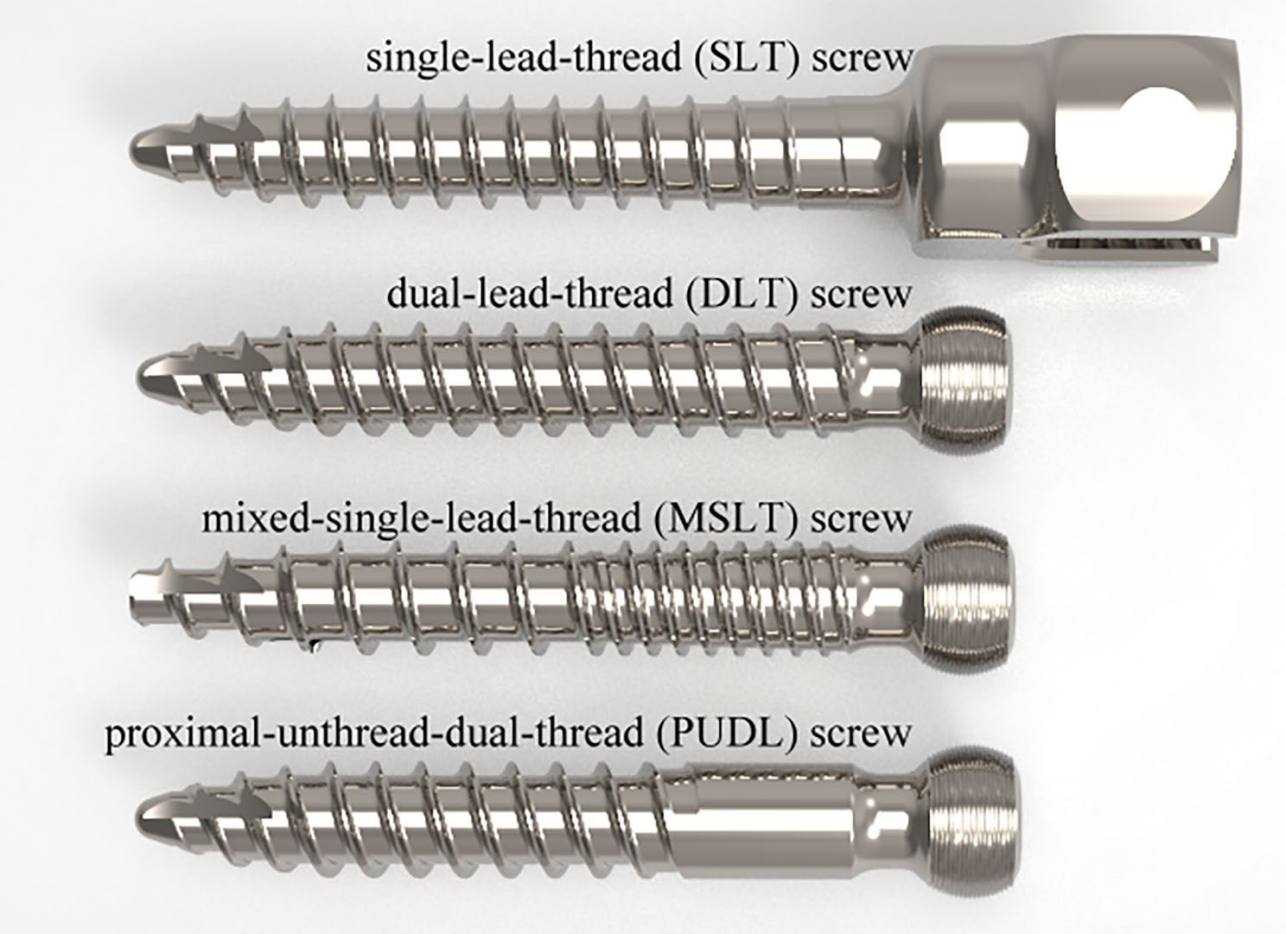
Pedicle screw designs evaluated in this study. SLT, DLT and MSLT screws are commercially available pedicle screws and the PUDL screw is a prototype design developed by the authors

Three different types of commercial pedicle screws (OCTOPODA, Bricon GmbH), and one prototype which was designed explicitly for use in this study by the investigators and manufactured by Bricon GmbH, were compared in an axial pullout test (Fig. 1). All screws were made of a titanium alloy (Ti6Al4V ELI) and had a screw shaft 35 mm long. The dimensions of the thread from the four screw designs are summarized in Table 1. The key difference between the pedicle screws tested was the thread design; single lead thread (SLT) screw, dual lead thread (DLT) screw, mixed single lead thread (MSLT) screw, and proximal unthreaded dual thread (PUDL) screw. The thread on the SLT screw was arranged in a single helical fashion along the entire core of the screw, whereas the threads on the DLT screw were arranged in a double-helical fashion. The thread on the MSLT screw was arranged in a single helical fashion along the proximal core of the screw, making the overall pitch narrower than the distal core. The PUDL screws were unthreaded for the initial 12 mm on the proximal end and had a double-helical thread along the remainder of the screw.

**Table 1:** Thread dimensions for the four pedicle screw designs

Screw Design	OD (mm)	ID (mm)	Pitch (mm)	Flank Angle (^o^)
SLT	6.0	4.4	2.5	10
	6.5	4.6		
DLT	6.0	4.4	2.5	20
	6.5	4.6		
MSLT	6.0	4.4	1.55/3.1 proximal/distal	10
	6.5	4.6		
PUDL	6.0	4.4	2.5	20
	6.5	4.6		

### Pullout strength testing

**Fig. 2:**
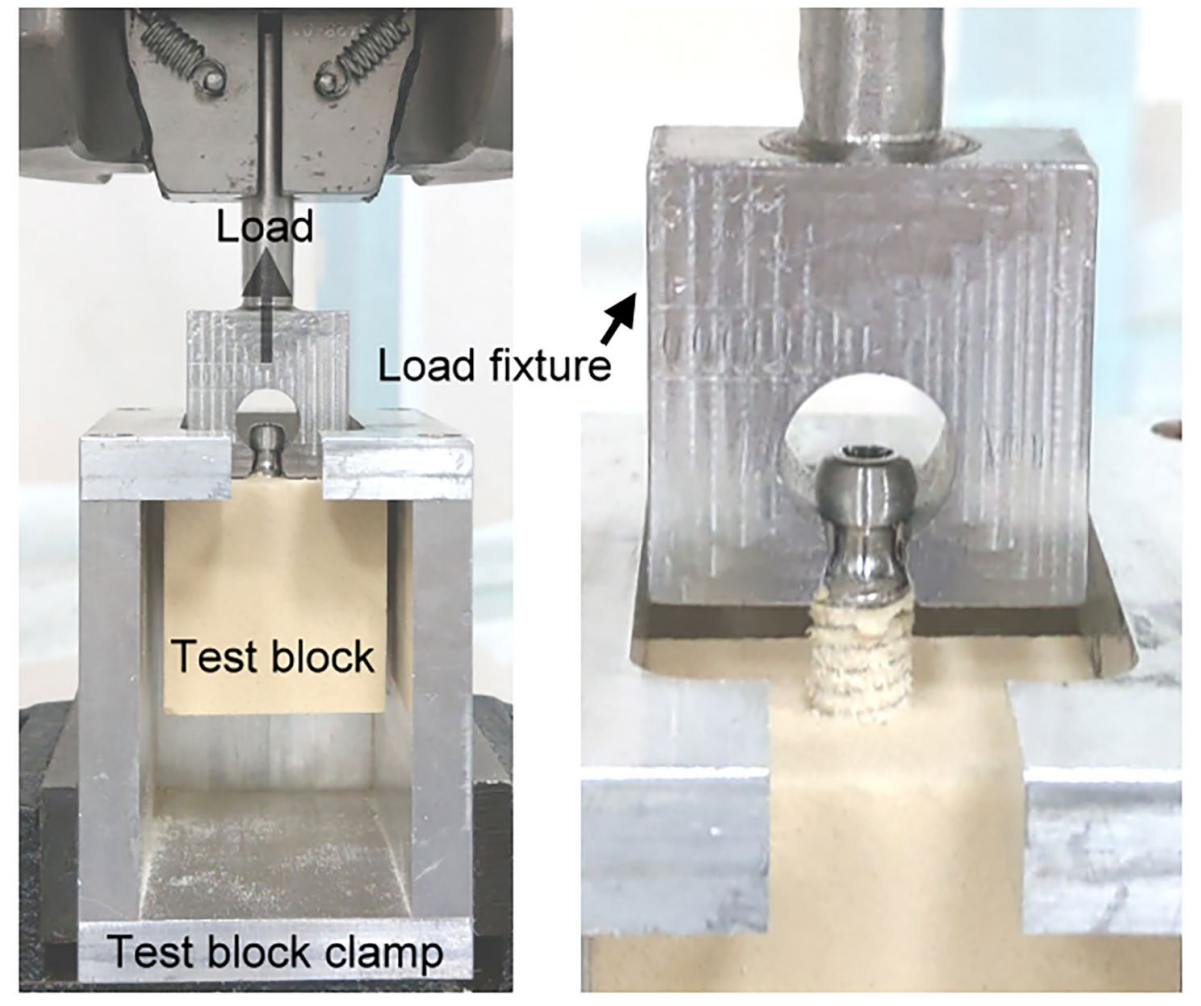
A custom-made pulling jig was used to perform the pullout strength test

An MTS MiniBionix testing system (MTS Systems Corporation, Eden Prairie, MN, USA) equipped with an MTS axial/torsional load cell (model 662.20 H-05) and a custom-made pulling jig was used to perform the pullout strength test according to ASTM F543 [14] (Fig. 2). The machine had an axial force capability of 25 kN and torque capacity of 250 Nm. A single hole was predrilled in each prepared solid foam block using a 4.2 mm diameter drill bit. The holes were drilled along the longitudinal axis of the pedicle screw, perpendicular to the surface of the foam. A pedicle screw was inserted into each hole to a depth of 30 mm at a rate of 3 rev/min.

The pull-test was performed by pulling the screw away from the test block at a rate of 1 mm/s. The fixation strength of each screw was defined by the maximum load recorded prior to failure by any means. Each of the four screw designs was tested five times using separate screws and separate foam blocks for each test.

Test Sequence.

**Fig. 3:**
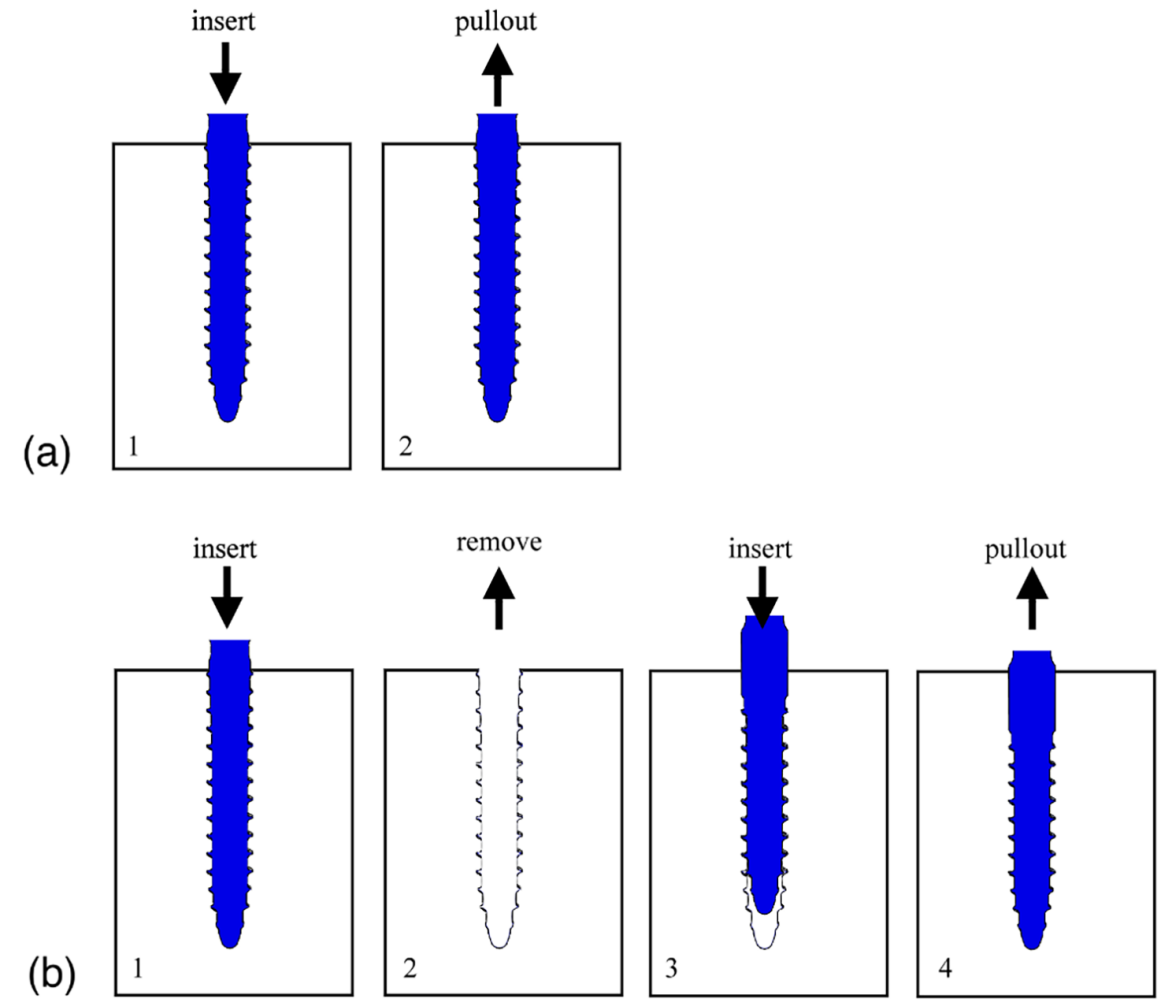
**A**) First sequence: Primary insertion and pullout test using the same screw; **B**) Second sequence: Primary insertion of a 6.0 mm diameter screw, followed by insertion of either a larger 6.5 mm screw of the same design or a 6.0 mm screw of a different design

In the first sequence, a 6.0 mm diameter screw was inserted into the predrilled foam block and the primary pullout strength of the screw was recorded (Fig. 3A).

In the second sequence, a 6.0 mm screw was inserted and removed, and then a new screw was inserted which was either (i) a 6.5 mm diameter screw of the same design, or (ii) a different screw design with a diameter of 6.0 mm. The pullout strength of the revision screw was recorded as the ‘revision pullout strength’ (Fig. 3B).

Each sequence was conducted five times for each screw design and the mean pullout strength and standard deviation were calculated.

Fisher’s post hoc test with a level of significance of 0.05 was used to ascertain significant differences between individual means when the analysis of variance identified significant differences.

## Results

**Fig. 4:**
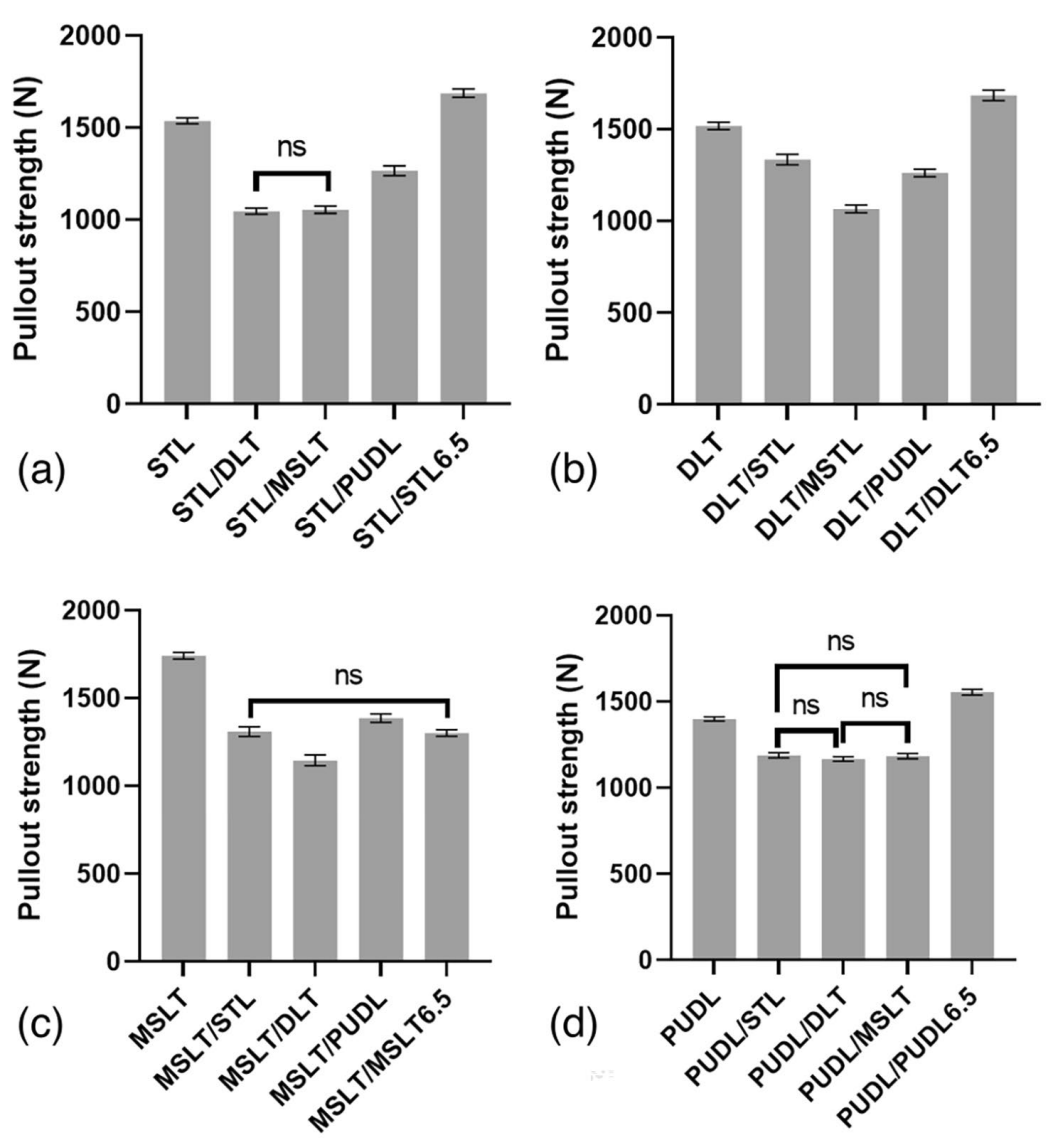
Mean pullout strength for the first and second sequence with different screw types. The bar graph shows differences in results that are not significant (ns) and the error bars represent plus or minus one standard deviation

In the first sequence, the mean pullout strength of MSLT was significantly (p < 0.05) greater than all other screw designs (Table 2). The difference in mean pullout strength between STL and DLT was not significant (ns, P > 0.05). In the second sequence, with STL or MSLT as the primary screw, using a PUDL screw for the revision resulted in a significantly greater pullout strength (p < 0.05) than the other screw designs. In addition, using a larger PUDL screw (6.5 mm) as the revision screw resulted in a higher pullout strength than using a 6.0 mm STL, DLT or MSLT screw for the revision. Similarly, using MSLT as the primary screw and a larger MSLT screw (6.5 mm) for the revision did not result in a higher pullout strength than the 6.0 mm diameter PUDL screw. Using a larger DLT screw (6.5 mm) as the revision screw was found to produce a significantly greater pullout strength than the 6.0 mm STL, DLT, MSLT, and PUDL screws. Similarly, when the primary screw was STL, only a larger STL screw (6.5 mm) resulted in a higher pullout strength for the revision. A comparison of the mean pullout strength of the different screw types is shown in Fig. 4.

**Table 2:** Pullout strength of the first and second sequence with different screw types

Type of primary screw	Pullout strength (N) of the first sequence	Pullout strength (N) of the second sequence Type of revision screw				
		STL	DLT	MSLT	PUDL	6.5 mm diameter. Same screw design
STL	1,538.3 ± 13.86		1,047.8 ± 14.47	1,056.4 ± 17.94	1,267.4 ± 24.34	1,688.0 ± 20.44
DLT	1,519.0 ± 18.19	1,336.6 ± 25.35		1,066.4 ± 18.91	1,263.4 ± 19.02	1,685.4 ± 25.07
MSLT	1,742.6 ± 16.01	1,310.4 ± 24.86	1,146.6 ± 27.40		1,387.0 ± 21.83	1,302.4 ± 16.89
PUDL	1,399.6 ± 10.80	1,188.4 ± 13.37	1,168.0 ± 11.82	1,184.8 ± 14.12		1,555.6 ± 14.76

## Discussion

The pullout testing of pedicle screws in study was performed by applying a gradual axial force at a constant displacement to a screw inserted into a block of synthetic bone, and the maximum force required to extract the screw was recorded as the pullout strength. Although such axial loading does not replicate the actual conditions in the body, the simplicity and reproducibility of the test method makes it an efficient method of investigating a screw’s holding capacity [15, 16]. Our previous study [9] showed that the pullout strength and withdrawal energy of a pedicle screw with the proximal 1/3 unthreaded inserted in osteoporotic Sawbone were greater than a fully threaded screw. This was the basis for the design of the PUDL screw in this current study. Few studies have reported on the strength of the fixation when the primary screw is replaced with a different screw design for the revision surgery. The results of this study compare the pullout strength of different types of pedicle screws when the revision screw is inserted in the same hole as the original screw.

In our study, the MSLT group had the greatest pullout strength for the primary fixation, and the PUDL group had the lowest. This is likely because the MSLT screw had more threads than the other designs and the bone-screw interface is a critical factor affecting the screw purchase in bone. There was no statistical difference in the primary pullout strength of the STL and DLT groups, but the difference in the remaining groups was significant (p < 0.05). A biomechanical study by Shen et al. [17] indicated that mixed-single-lead-thread screws have a larger bone-screw contact interface, indicating that such screws would have a greater pullout strength. In contrast, the effective bone-screw interface of the PUDL screw where the proximal 1/3 does not have a thread would be less than the other groups, and hence the pullout strength is approximately 10% lower than the fully threaded screws (STL). The relationship between holding strength and the area of the effective bone-screw interface is supported by the similar results for the STL and DLT groups, where both screws have a comparable bone-screw interface and similar pullout strength.

**Fig. 5:**
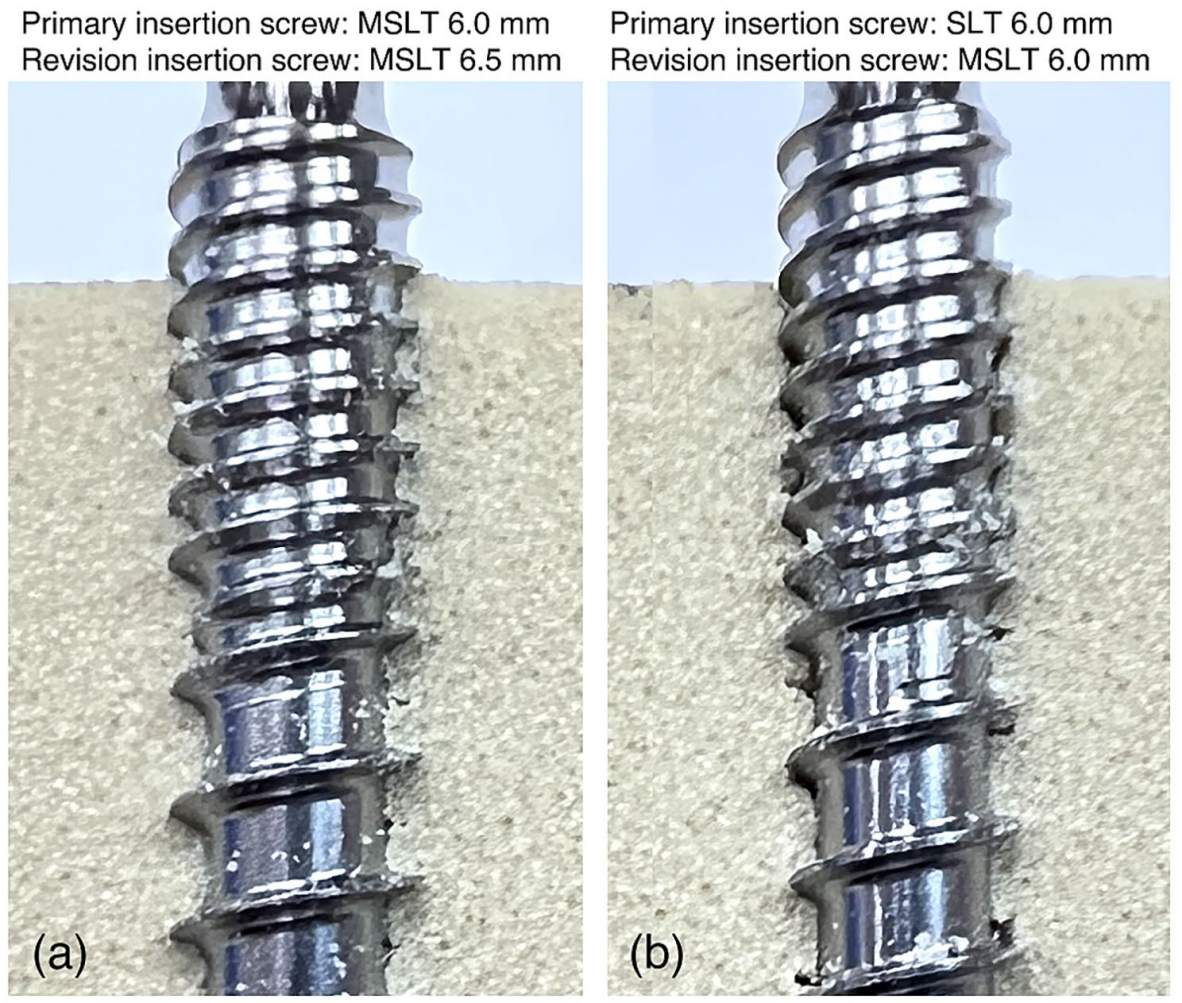
Observation of bone defects when using the MSLT and STL screws

When the same size screw with a different thread was used for the revision (second sequence), the pullout strength for all revision screws was lower than when the primary screw was replaced by the same screw of larger diameter (second sequence). When an STL or MSLT screw was used for the primary insertion, inserting a PUDL screw for the revision resulted in the greatest pullout strength of all groups in the second sequence. If a DLT or PUDL was used for the primary insertion, the STL screw had the greatest pullout strength when used for the revision. The results show that the revision pullout strength in the second sequence was lowest when using a DLT or MSLT screw. This is likely because the insertion path was already tapped by the primary screw and the insertion hole was more severely damaged by the dual-thread or mixed-thread screw in the revision procedure. After inserting a DLT or MSLT screw for the revision, there was noticeable damage to the Sawbone in the MSLT group at the bone-screw contact interface (Fig. 5), which is similar to results from a previous biomechanical study on pullout testing of dual-thread screws [18]. Hence, we considered that dual-thread or mixed-thread screws may be used for primary fixation or when the bone is of good quality, but may not be suitable as revision screws. A biomechanical study by Tsuang et al. [9] reported PUDL screws with excellent pullout strength and withdrawal energy in osteoporotic bone. In this current study, the PUDL screw in the second insertion had a similar pullout strength to the STL screws. We believe that the damaged bone structure during the second insertion could be regarded as representative of osteoporotic bone, and the pullout strength of the PUDL screw was greater than or equal to the other screw types.

Increasing the diameter of the revision screw improves the purchase in the bone, forcing the thread to engage in the bone around the existing tunnel [3, 10]. Seng et al. [10] showed that revising a 6.5 mm pedicle screw with a 7.5 mm screw resulted in a greater revision pullout strength than if the revision was performed with a 6.5 mm screw. Similarly, this current study found that the pullout strength of the larger diameter revision screw was higher than the primary pullout strength of the smaller screw, except for with the MSLT screw. The apparently lower pullout strength of the larger diameter MSLT screw is likely because of the poor bone quality at the bone-screw interface which was damaged during the removal of the screw with a different thread design on the proximal and distal ends (Fig. 5a) [10, 19]. In contrast, the other three screws have a more uniform thread pitch. The pitch, thread angle, thread depth, and helix angle were identical for the 6.0 and 6.5 mm diameter screws of the same design. Our results suggest that a classic oversizing approach with the same screw design can maintain adequate screw purchase when replacing the primary screw. If oversizing with the same screw design is not feasible, inserting a PUDL screw with the same diameter as the primary screw provides the greatest pullout strength.

There are some limitations to this study. Firstly, the synthetic bone blocks used are designed to replicate the properties of bone, but the nonlinear properties of human bone cannot be truly represented by such synthetic materials. However, synthetic blocks are routinely used in biomechanical studies because they are readily available, are cheap and display low interspecimen variability. Second, the pullout strength of the screws was only tested under an axial force, which does not represent the loading conditions in the body or the forces placed on pedicle screws. Third, we restricted our study to four common screw types with defined diameters and excluded other pedicle screw designs. The results may not be representative of all screw types. Last, this study only considered the situation where the primary screw was removed and a secondary screw reinserted into the same hole without considering other aggravating factors that may lead to screw loosening, such as screw hole enlargement. Future work may investigate the fatigue properties of the different screw insertions under cyclic loading.

## Conclusion

The results of this study show that a classic oversizing approach with the same screw design is the best option for a revision screw. If oversizing with the same screw design could is not feasible, using a PUDL screw with same original diameter can provide adequate purchase in the bone to maintain stability.

## Data Availability

The datasets used and/or analyzed during the current study are available from the corresponding author upon reasonable request.

## Abbreviations

SLT: Single-lead-thread
DLT: Dual-lead-thread
MSLT: Mixed-single-lead-thread
PUDL: Proximal-unthread-dual-thread
PMMA: Polymethylmethacrylate
